# Intracranial Epstein-Barr virus-associated smooth muscle tumor with superimposed cryptococcal infection: A case report

**DOI:** 10.1097/MD.0000000000028806

**Published:** 2022-03-04

**Authors:** Kaylyn Barrett, Sam Tavakoli, Michael McGinity, Andrea Gilbert

**Affiliations:** a University of Texas Health San Antonio Long School of Medicine, TX; b University of Texas Health San Antonio Department of Neurosurgery, TX; c University of Texas Health San Antonio Department of Pathology and Laboratory Medicine, TX.

**Keywords:** case report, CNS, cryptococcus, EBV-SMT, Epstein-Barr virus associated smooth muscle tumor, intracranial

## Abstract

**Rationale:**

Epstein-Barr virus-associated smooth muscle tumors (EBV-SMT) are rare, virally-induced malignancies that occur almost exclusively in immunocompromised individuals. We report a very rare case of a dura-based EBV-SMT with superimposed local cryptococcal infection.

**Patient concerns:**

An adult male with a history of untreated acquired immunodeficiency syndrome presented to our hospital with worsening headaches, diarrhea, and diffuse myalgias.

**Diagnoses:**

Blood cultures were positive for methicillin-resistant *Staphylococcus aureus* and *Cryptococcus neoformans* serum antigen. Magnetic resonance imaging revealed 2 adjacent enhancing masses in the right temporal lobe, perilesional edema, and mass effect of the right lateral ventricle. Histological examination and immunohistochemical stains of the surgical specimen were consistent with EBV-SMT. Cryptococcus organisms were identified within the neoplasm.

**Interventions:**

The patient underwent complete tumor resection, received an extended course of amphotericin and flucytosine, and was restarted on antiretroviral therapy.

**Outcomes:**

The patient was discharged from the hospital with no focal neurological deficits.

**Lessons:**

Epstein-Barr virus associated smooth muscle tumors are rare malignancies that occur in immunocompromised patients. Prognosis is largely dependent on immune reconstitution and treatment of concomitant infections.

## 1 Introduction

Epstein-Barr virus associated-smooth muscle tumor (EBV-SMT) is a rare, virally induced malignancy that occurs almost exclusively in immunocompromised individuals. They are most commonly described in persons infected with human immunodeficiency virus (HIV) or treated with immunosuppressive agents following organ transplant. EBV-SMTs are histologically distinct from classic smooth muscle tumors. In contrast to classic smooth muscle tumors, EBV-SMT histology does not correlate well with clinical outcomes.^[1]^ In addition to the roughly 65 cases of central nervous system (CNS) tumors documented in the literature,^[2]^ EBV-SMTs have been described in a variety of anatomic locations, including the liver, lungs, adrenal glands, gastrointestinal tract, and genitourinary organs.^[3,4]^

Opportunistic infections are common in patients with acquired immunodeficiency syndrome (AIDS), but their concomitant incidence with EBV-SMT is not well documented. To our knowledge, we describe the first report of a cryptococcal intracranial infection superimposed on a dural EBV-SMT in an HIV-positive patient.

## 2 Case report

A 42-year old male with a several-year history of AIDS, untreated within the last year, and a new diagnosis of a brain mass 3 months prior presented to our institution with worsening headaches, diarrhea, and diffuse myalgias. Previous workup did not detect infection with Coccidiodes, Histoplasma, Cryptococcus, or Human Polyoma Virus 2. Physical examination did not reveal any focal neurological deficits. On admission, his CD4 count was <40 cells/mL (normal >500 cells/mL) and viral load was 20,900 copies/mL. Blood culture was positive for methicillin-resistant *Staphylococcus aureus* (MRSA) and *Cryptococcus neoformans* serum antigen; therefore, intravenous antibiotics and antifungals were initiated soon after admission.

Magnetic resonance imaging revealed a 5 cm enhancing and necrotic mass in the right temporal lobe with perilesional vasogenic edema causing effacement of the adjacent right hemispheric sulci, mass effect on the right lateral ventricle, and a 5 mm right to left midline shift. An adjacent 1.5 cm lesion was also observed. Given the patient's HIV-associated immunocompromised state and cryptococcemia, the differential for these intracerebral lesions included lymphoma, cryptococcoma, brain abscess, syphilitic gumma, tuberculoma, and toxoplasmosis.

The tumor was resected via open craniotomy using standard neurosurgical techniques. A small amount of fluid present in the center of the tumor was cultured. The tumor was microscopically dissected from its tentorial attachment at the transverse sigmoid junction and, after the entire tumor was resected, hemostasis was obtained. Standard closure was performed.

**Figure 1:**
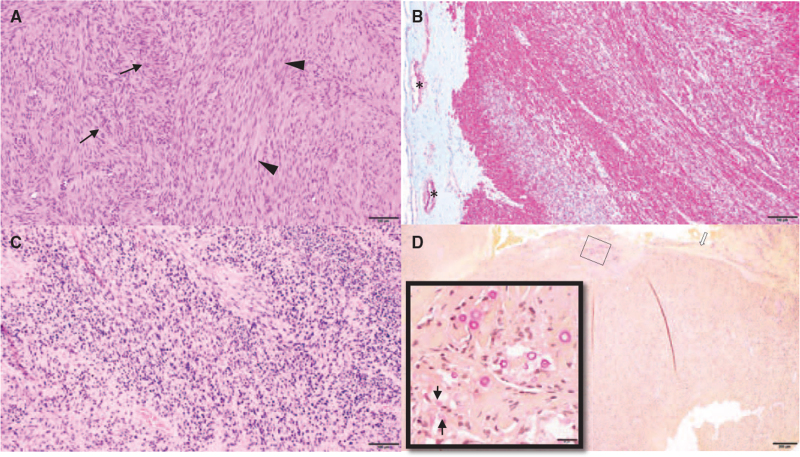
EBV-SMT histology. A. Hematoxylin and eosin of smooth muscle morphology featuring cells with spindled nuclei and syncytial cytoplasm arranged in long intersecting fascicles cut in cross-section (arrows) and longitudinal section (arrowheads) (magnification: 100×). B. SMA red chromagen highlights smooth muscle differentiation in the tumor, as well as smooth muscle of dural arteries (asterisk) (magnification: 100×). C. EBER ISH staining blue EBV-infected tumor nuclei (magnification: 100×). D. Mucicarmine stain highlighting fungal organisms morphologically compatible with Cryptococcus with narrow-based budding (black arrows) located within the tumor adjacent to the dural attachment (white arrow) (magnification: 40×; inset: 400×).

Microscopic sections stained with hematoxylin and eosin showed a neoplasm with smooth muscle morphology, featuring a relatively monomorphic population of compact spindle cells arranged in long intersecting fascicles (Fig. 1A). Proliferative activity was elevated with the most active region showing 7 mitoses seen in 10 high-power fields on hematoxylin and eosin staining. Immunohistochemical analysis showed that tumor cells were positive for muscle markers, including desmin and smooth muscle actin (Fig. 1B), but were negative for EMA, PR, and STAT6, thereby effectively ruling out other entities in the differential, including meningioma and solitary fibrous tumor (hemangiopericytoma). EBV-encoded RNA evaluation by in situ hybridization showed strong positive detection of EBV RNA in the nuclei of almost all tumor cells (Fig. 1C). In addition, also present within the spindle cell neoplasm were small yeast organisms (average diameter: 4-6 μm) with narrow-neck budding and strong capsular staining with mucicarmine, morphologically compatible with Cryptococcus (Fig. 1D). A diagnosis of EBV-SMT with concomitant cryptococcal infection was rendered.

The patient awoke from surgery without any neurological deficits, and postoperative imaging demonstrated gross total resection. The patient later complained of persistent headaches. Lumbar puncture was performed on postoperative day (POD) 7, which revealed an opening pressure of 28 mm Hg for which a high-volume tap was performed. Due to continued symptoms of increased intracranial pressure, repeated lumbar punctures were performed on PODs 14 and 20 with opening pressures of 17 mm Hg and 28 mm Hg, respectively. After an unsuccessful attempt at lumbar puncture on POD 25 the patient refused any additional attempts despite continued symptoms.

His hospital course was complicated by persistent MRSA bacteremia, femoral osteomyelitis, and acute pyelonephritis secondary to MRSA bacteremia. Amphotericin and vancomycin were continued postoperatively, and flucytosine was added to the regimen on POD 5. Anti-retroviral therapy was held until POD 17 due to concerns regarding the risk of immune reconstitution inflammatory syndrome with active CNS cryptococcal infection. The patient was discharged home on POD 51 and he did not present for further follow-up.

## 3 Discussion

EBV-SMTs are rare neoplasms that may arise in a variety of tissues and organs in persons with immunodeficiency, such as HIV-infected individuals, or immunosuppression, such as in transplant patients. Although the mechanism of EBV-induced neoplastic transformation of smooth muscle cells is still under investigation, the observation of small EBV-SMTs occurring around blood vessels has led some authors to suggest a possible viral tropism for vascular smooth muscle ^[3,5]^ and novel expression of the EBV receptor on smooth muscle cells of AIDS patients has been hypothesized as a likely mechanism for viral entry.^[1]^

At the time of diagnosis, approximately half of all patients with EBV-SMTs have multiple lesions, which are almost always multifocal.^[4]^ Distinct lesions are thought to represent separate infection events by different EBV clones, rather than metastases of a primary lesion.^[1]^ Because of this multifocal nature, immune reconstitution is highly recommended to reduce further opportunities for EBV-induced neoplastic transformation of smooth muscle tissue.

In the setting of HIV, EBV-SMT is most frequently found in the CNS and surgical excision is the mainstay of treatment for these tumors.^[3]^ Total resection is recommended in patients with a mass effect, as occurred in our case, or focal deficits. A greater extent of resection is associated with a survival benefit, particularly in those with intracranial invasion, likely due to the relief of the mass effect or cranial nerve dysfunction.^[2]^ Adjunctive treatment options include radiotherapy, which is more often used in HIV/AIDS patients, or chemotherapy, which is typically used in postoperative transplant patients. In our case, intravenous antifungal agents were also used for the treatment of CNS cryptococcal infection and cryptococcemia.

Understanding the natural history of EBV-SMT is complicated by the common occurrence of opportunistic infections in this population. In as many as 90% of patients with EBV-SMT, death is secondary to opportunistic infections rather than tumor burden.^[4,6]^ There are few reports of disseminated cryptococcal infection or cryptococcal meningitis occurring concomitantly in a patient with EBV-SMT(s) of the CNS.^[7–9]^ To our knowledge, there has not been any previously described cases of EBV-SMT with infectious organisms present within the tumor matrix, such as seen in this case.

## 4 Conclusion

We report a case of a dura-based EBV-SMT with superimposed local cryptococcal infection, which, to our knowledge, has not been previously described in the literature. Despite the relatively indolent course of extracranial EBV-SMT, surgical intervention with an attempt for maximal total resection is recommended for individuals with mass effect or focal CNS deficits. Immune reconstitution and aggressive treatment for concomitant infections are paramount. Despite its rarity, EBV-SMT should be considered in immunodeficient or immunosuppressed patients presenting with CNS mass.

## Author contributions

The authors [Kaylyn Barrett, BS; Samon Tavakoli, MD; Michael McGinity, MD; Andrea Gilbert, DO] organized the order of the visits of this patient and interpreted the results. All authors approved the final manuscript as submitted and agree to be accountable for all aspects of the work.

**Conceptualization:** Sam Tavakoli, Michael McGinity, Andrea Gilbert.

**Data curation:** Sam Tavakoli.

**Investigation:** Sam Tavakoli.

**Project administration:** Michael McGinity, Andrea Gilbert.

**Supervision:** Michael McGinity.

**Writing – original draft:** Kaylyn Rose Barrett.

**Writing – review & editing:** Kaylyn Rose Barrett, Sam Tavakoli, Andrea Gilbert.
